# INTERSECTION OF RACE/ETHNICITY, NATIVITY, AND EMPLOYMENT IN SLEEP QUALITY: A MULTILEVEL MODELING STUDY

**DOI:** 10.1093/geroni/igae098.0780

**Published:** 2024-12-31

**Authors:** Maki Karakida, Jeffrey Stokes

**Affiliations:** University of Massachusetts Boston, UMass Boston, Massachusetts, United States; University of Massachusetts Boston, Boston, Massachusetts, United States

## Abstract

Sleep disorder is a common problem seen in middle-aged and older adults. Work-related stress can also impact people’s sleep quality, yet differences between working and retired older adults’ sleep is unclear. This study examines the relationships between work, retirement, and sleep health, including their intersection with race/ethnicity and nativity, among 31,868 Americans aged 50+. Using the Health and Retirement Study samples (2014 – 2020), we analyzed how diverse older workers and retirees experienced their sleep health differently. We used multilevel linear regression to examine the associations between working/retirement status, race/ethnicity, birthplace, and sleeplessness, controlling for age, gender, birthplace, assets, education, marital status, smoking, exercise, and self-reported health. The regression model without interaction terms demonstrated that compared to retirees, older workers have a significantly higher likelihood of sleep disorder. Black race, assets, education, and age had positive and significant relationships with sleep deprivation, whereas martial loss (divorced, widowed, and never-married) and female gender had significantly lower onsets of sleep disturbances. In the model examining the intersection of race/ethnicity and nativity - interacted with work status - revealed that, compared with US-born non-Hispanic White adults, US-born and foreign-born non-Hispanic Black adults had a significantly higher risk of insomnia. In contrast, non-native Hispanic workers and foreign-born non-Hispanic Other workers had a significantly higher prevalence of sleep difficulties than US-born non-Hispanic White workers. Multilevel findings suggest that the intersectionality of race/ethnicity and nativity plays a decisive role in the association of work status and sleep quality among midlife and older adults in the US.

